# Reply to: Complementary of modified NUTRIC score with or without C-reactive protein and subjective global assessment in predicting mortality in critically ill patients

**DOI:** 10.5935/0103-507X.20210022

**Published:** 2021

**Authors:** Manoela Lima Oliveira, Daren Keith Heyland, Flávia Moraes Silva, Estela Iraci Rabito, Mariane Rosa, Micheli da Silva Tarnowski, Daieni Fernandes, Aline Marcadenti

**Affiliations:** 1 Universidade Federal de Ciências da Saúde de Porto Alegre - Porto Alegre (RS), Brazil.; 2 Clinical Evaluation Research Unit, Kingston General Hospital - Kingston, Ontario, Canada.; 3 Department of Public Health, Queen’s University - Kingston, Ontario, Canada.; 4 Department of Critical Care Medicine, Queen’s University - Kingston, Ontario, Canada.; 5 Department of Nutrition, Universidade Federal de Ciências da Saúde de Porto Alegre - Porto Alegre (RS), Brazil.; 6 Postgraduate Program in Nutrition Sciences, Universidade Federal de Ciências da Saúde de Porto Alegre - Porto Alegre (RS), Brazil.; 7 Postgraduate Program in Food and Nutrition, Universidade Federal do Paraná - Curitiba (PR), Brazil.; 8 Division of Nutrition, Santa Casa de Misericórdia de Porto Alegre - Porto Alegre (RS), Brazil.; 9 Research Institute, HCor-Hospital do Coração - São Paulo (SP), Brazil.; 10 Postgraduate Program in Health Sciences: Cardiology, Instituto de Cardiologia do Rio Grande do Sul - Porto Alegre (RS), Brazil.

**TO THE EDITOR**

We revised the letter to the Editor about our study with high interest.^(1)^ Please, find below are our comments.

Regarding the first comment, we agree that our sample was limited, and our results come from a single-center study; indeed, this was remarked in our manuscript as a limitation. As we stated in Methods, we excluded patients at imminent risk of death, which might have influenced our results. Our team decided to exclude those patients for ethical concerns added to the challenge of collecting a proxy signature on the informed consent form. That would be contrary both to the Ethics Regulation from the Ethics Committee of the *Universidade Federal de Ciências da Saúde de Porto Alegre* and the Ethics Committee of the *Irmandade da Santa Casa de Porto Alegre*, where the data were collected. We agree that more studies are necessary to either confirm or challenge our hypothesis.

Regarding the second comment, we agree that C-reactive protein (CRP) is widely used as a biomarker in intensive care units (ICU), and that every patient admitted to an ICU would be expected to have routine CRP results. However, this is not true in Brazil, particularly in the Hospital where our research was conducted. We aimed to use the Nutrition Risk in the Critically Ill (NUTRIC) score as originally proposed: a nutritional assessment tool; for this, it would have to be completed within 48 hours of the patient’s admission. As CRP results for some subjects were not evaluable by this time, our second goal was to use the NUTRIC score as an assessment tool that could be widely available in Brazilian ICUs and did not want that missing CRP results represented an exclusion criterion for using the NUTRIC score. Therefore, we decided to compare the NUTRIC-CRP and the modified NUTRIC (mNUTRIC) scores. In our study the agreement between mNUTRIC and NUTRIC-CRP was found to be excellent (n = 90; Kappa = 0.88, p < 0.001). Additionally, acute inflammation markers as CRP or interleukin 6 were excluded from the original NUTRIC score because their inclusion failed to improve the final model fit, and the score was not improved when these variables were added.^(2)^

We believe that, in settings where CRP values are available, adding them to the NUTRIC score could be helpful; however, we also believe that, when this or any other inflammatory biomarker is not available in the clinical practice, this assessment tool would remain useful.
